# Toxicogenomic Analysis Suggests Chemical-Induced Sexual Dimorphism in the Expression of Metabolic Genes in Zebrafish Liver

**DOI:** 10.1371/journal.pone.0051971

**Published:** 2012-12-18

**Authors:** Xun Zhang, Choong Yong Ung, Siew Hong Lam, Jing Ma, Yu Zong Chen, Louxin Zhang, Zhiyuan Gong, Baowen Li

**Affiliations:** 1 NUS Graduate School for Integrative Sciences and Engineering, National University of Singapore, Singapore, Singapore; 2 Centre for Computational Science and Engineering, National University of Singapore, Singapore, Singapore; 3 Department of Biological Sciences, National University of Singapore, Singapore, Singapore; 4 Department of Mathematics, National University of Singapore, Singapore, Singapore; 5 Department of Pharmacy, National University of Singapore, Singapore, Singapore; 6 Department of Physics, National University of Singapore, Singapore, Singapore; The Centre for Research and Technology, Hellas, Greece

## Abstract

Differential gene expression in two sexes is widespread throughout the animal kingdom, giving rise to sex-dimorphic gene activities and sex-dependent adaptability to environmental cues, diets, growth and development as well as susceptibility to diseases. Here, we present a study using a toxicogenomic approach to investigate metabolic genes that show sex-dimorphic expression in the zebrafish liver triggered by several chemicals. Our analysis revealed that, besides the known genes for xenobiotic metabolism, many functionally diverse metabolic genes, such as ELOVL fatty acid elongase, DNA-directed RNA polymerase, and hydroxysteroid dehydrogenase, were also sex-dimorphic in their response to chemical treatments. Moreover, sex-dimorphic responses were also observed at the pathway level. Pathways belonging to xenobiotic metabolism, lipid metabolism, and nucleotide metabolism were enriched with sex-dimorphically expressed genes. We also observed temporal differences of the sex-dimorphic responses, suggesting that both genes and pathways are differently correlated during different periods of chemical perturbation. The ubiquity of sex-dimorphic activities at different biological hierarchies indicate the importance and the need of considering the sex factor in many areas of biological researches, especially in toxicology and pathology.

## Introduction

Sexual dimorphism occurs at various biological levels throughout the life span of the organisms that reproduce sexually, where males and females show obvious anatomical, physiological, and behavioral differences. Due to the different strategies adopted to maximize survival fitness, exogenous perturbations could trigger observable disparity of endogenous alterations that possibly link to phenotypic variations in opposite sexes. This is evident by the increasing reports that males and females differ in susceptibility to diseases, vulnerability to pharmaceuticals in terms of drug efficacy and adverse drug reactions, as well as differential responsiveness to various xenobiotics [1]–[3].

The sex-dependent responses to exogenous perturbations are deemed to originate from discrepant cellular milieus and homeostasis in male and female. Traditionally, it is assumed that the cause of these differences is primarily due to the secretion of gonadal hormones [4]–[6]. Therefore, hormonal regulation in differentiation and development of gonadal and non-gonadal organs has been the focus of scientific research [7]–[10]. Recently, researchers have observed that a large number of genes exhibited profound sexual differences at the transcriptomic level in non-gonadal tissues such as brain and liver [11]–[19]. These reports collectively suggest that sexual dimorphism stems from sex-dependent genetic and hormonal regulation, and is widespread across many, if not all, organs. Although the hormonal and transcriptomic differences between opposite sexes are well known under normal physiology, there is still limited information on sex-dependent gene expression in responding to exogenous perturbations [20].

The liver is the primary organ in mediating general metabolism and detoxification. A number of studies have shown that many hepatic genes associated with xenobiotic metabolism, such as cytochrome P450, are expressed in a sex-dimorphic manner during detoxification [15], [16], [21]. Apart from sex-dependent activities of genes related to xenobiotic metabolism, it is still unclear to what extent other forms of metabolic genes are differentially expressed in the two sexes in response to exogenous perturbations. As aberrant metabolic states in the opposite sexes are differentially associated with metabolic-related syndromes and diseases, such as obesity, type 2 diabetes, and cardiovascular diseases [22]–[25], chemical-induced sexual dimorphism in metabolic-related genes seem relevant.

In this work, we are interested in identifying metabolic genes and pathways that display sex-dimorphic activities induced by different chemicals. Here we used the zebrafish model treated with four different environmental toxicants: 4-chloroaniline, 4-nitrophenol, arsenic (V), and cadmium (II). Sex-dimorphic expression of metabolic genes was then examined from the microarray data derived from these chemical-treated fish. Genes that show sex-dependent responses in the same direction across all chemical treatment groups (i.e. up-regulated in male but down-regulated in female or vice versa) are considered to be sex-dimorphic. We found that there were common sex-dependent responses of metabolic genes and pathways under these four chemical perturbations. We also traced the dynamics of the sex-dependent responses from individual genes, to pathways and pathway networks at the metabolic module level. To our knowledge, this is the first study addressing chemical-induced sex-dimorphic expression of metabolic genes and pathways in zebrafish. Results in this work indicate the potential of using the zebrafish model to understand sex-dependent toxicology and pathology.

## Results and Discussion

### Hierarchical Clustering of Zebrafish Liver Metabolic Transcript Profiles Suggests Chemical-induced Sex-dimorphic Responses

**Figure 1:**
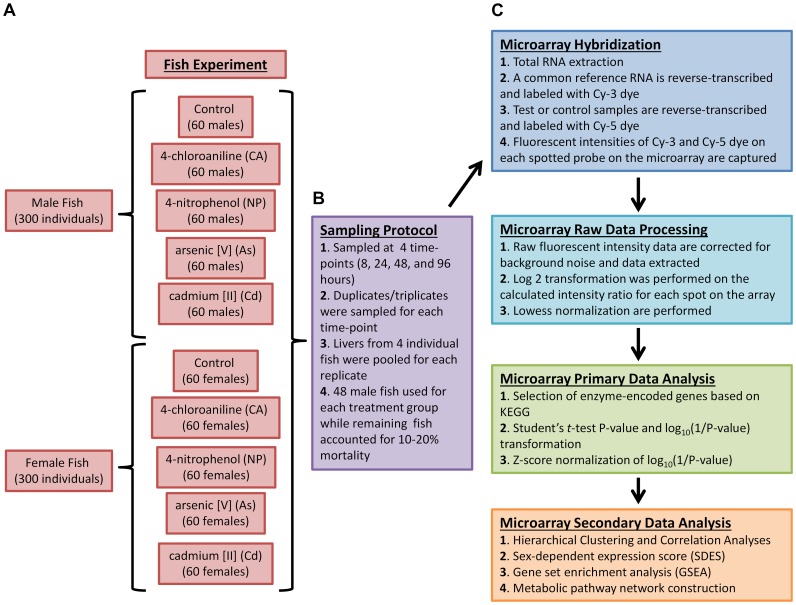
Flow chart of the overall experimental outline and microarray analysis. (A) Experimental design of chemical treatments. A total of 300 male and 300 female fish were used in the experiment involving 4 chemicals (CA, 4-chloroaniline; NP, 4-nitrophenol; As, arsenic (V); Cd, cadmium (II)) and a control (untreated) group, sampled at 4 different time points (8, 24, 48, 96 h). (B) Sampling protocol. Fish were maintained at a density of one fish per 200 ml and sampled in replicates (duplicates/triplicates) for each treatment time point. Excess fish were factored to account for 10–20% mortality during the experiment. Livers from 4 individual fish were pooled for each replicate (C) Microarray experiments and data analyses. Total RNAs extracted from the pooled liver were reverse-transcribed and labelled with Cy-5 dye, while a common reference RNA was labelled with Cy-3 dye for competitive hybridization with the probes spotted on the microarray. Fluorescent intensities of Cy-5 and Cy-3 dyes were recorded and raw data were normalized using Lowess method. Statistical test was performed for each treatment group followed by Z-score normalization for combined data for all treatments. Hierarchical clustering and correlation analyses for the identification of sex-dimorphic gene expression were conducted. Gene Set Enrichment Analysis (GSEA) was used to infer enriched sex-dependent metabolic pathways and their network correlation.

The overall experimental outline and microarray analysis are summarized in **Figure 1**. A total of 92 arrays for the chemical-treated groups (CA, 4-chloroaniline; NP, 4-nitrophenol; As, arsenic (V); Cd, cadmium (II)) at four time points (8 h, 24 h, 48 h, and 96 h) (duplicates for Cd female 8h, Cd female 96 h, Cd male 8 h, and Cd male 24 h; others are triplicates) and 24 arrays (triplicates for both male and female at 8 h, 24 h, 48 h, and 96 h) for the common control (untreated) groups were used. The full details of chemical concentrations, replicates, and the accession of the microarray data are given in **Table S1**. Annotated genes involve in a wide range of metabolism including xenobiotic metabolism are considered. Genes missing from microarray data under one or more chemical-treatment conditions were eliminated. Gene symbol for the zebrafish genes was obtained via mapping to human homologous genes and was subsequently used to map with metabolic genes defined in the Kyoto Encyclopedia of Genes and Genomes (KEGG) database [26]. A total of 307 metabolic genes were obtained under these selection criteria for further analysis (**Table S2**).

**Figure 2:**
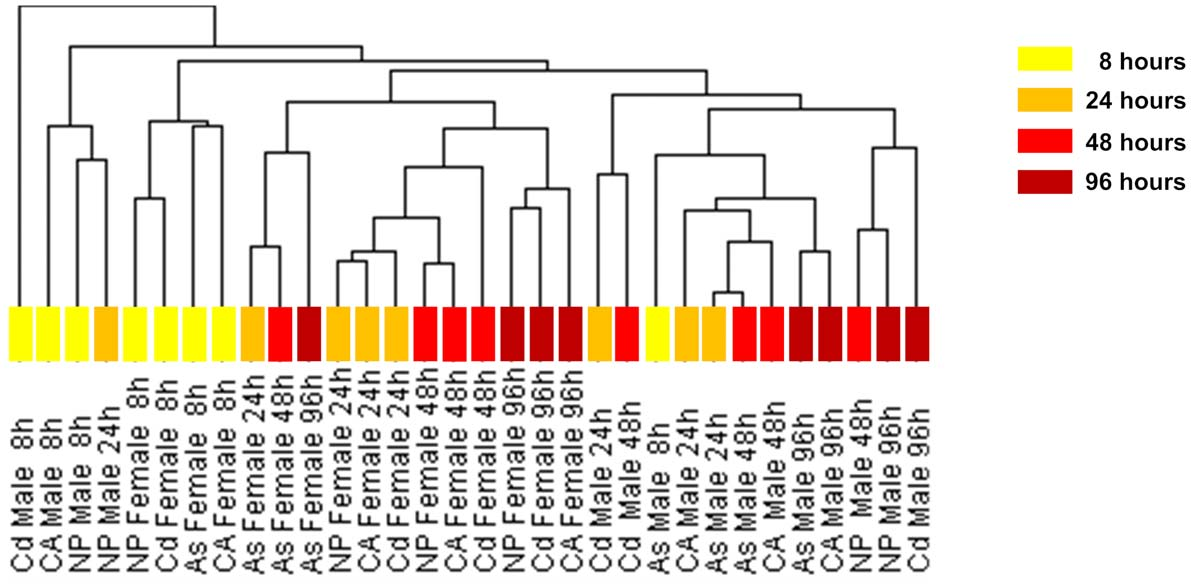
Hierarchical clustering of zebrafish liver metabolic transcript profiles in response to chemical perturbations. Unsupervised hierarchical clustering (Spearman rank correlation) of the metabolic transcript profiles indicates sex-dependent metabolic gene expression in response to chemical treatment. Branches placed under subtrees only consist of one sex. In general, branches clustered under subtrees belong to the same stage of chemical treatment.

**Figure 3:**
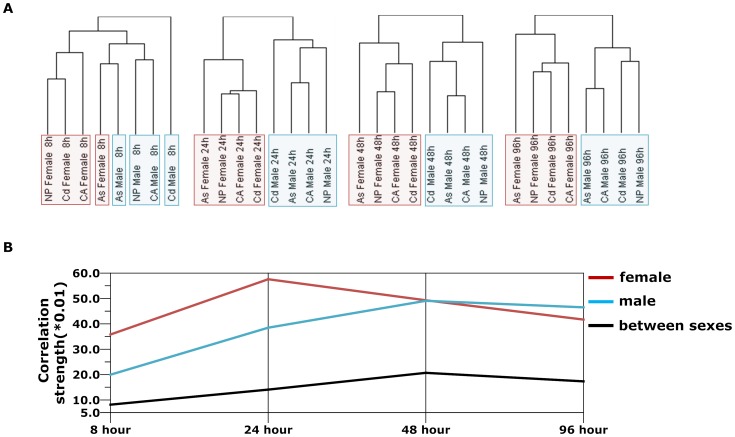
Hierarchical clustering of zebrafish liver metabolic transcript profiles at different time points in response to chemical perturbations. (A) Unsupervised hierarchical clustering (Spearman rank correlation) of metabolic transcript profiles indicates sex-dependent liver metabolic gene expression in response to chemical perturbation at different time points. (B) The correlation strength of metabolic transcript profiles within the same sex group changed dynamically during chemical perturbation. At different time points, the average correlation strength of each sex are calculated by averaging the Spearman rank correlation coefficients on any two metabolic transcript profiles for the respective sex. Variations of correlation strength on metabolic gene expression are represented by the blue curve for male and red curve for female. The correlation strength of metabolic transcript profiles between opposite sexes is represented by the black curve, which remained at a low level throughout the 96-hour chemical treatment.

Unsupervised hierarchical clustering was applied to all datasets to generate a dendrogram (**Figure 2**). It is obvious that branches under each subtree belong to one sex, either male or female. Moreover, the branches of the dendrogram were generally organized according to different treatment times, indicating the progression of chemical-induced sex-dimorphic responses. This observation led us to further interrogate the dynamics of metabolic response with respect to increasing duration of chemical treatment. We applied the unsupervised hierarchical clustering (Spearman rank correlation) to the eight metabolic transcript profiles (CA female & male, NP female & male, As female & male, Cd female & male) for each time point. At 8 hours of chemical treatment, with the exception of arsenic, transcript profiles of the three other chemical treatment groups were loosely clustered according to sex. The early impact of arsenic exposure on the metabolic gene expression appeared to surpass the effects of sex-dependency so that arsenic-treated males and females were clustered together. However, a clear sex-dependent clustering pattern was observed starting from 24 to 96 hours of chemical treatment (**Figure 3A**, details presented in **Figure S1**)**.** Similar clustering results were also obtained using Pearson correlation (**Figure S2**), indicating the consistency and robustness of the sex-dependent clustering patterns of the metabolic transcript profiles. **Figure 3B** displays the correlation strength of the clustering metabolic transcript profile within the same sex and between the opposite sexes that changed dynamically during the chemical perturbation period. Metabolic transcript profiles within the same sex group exhibited higher similarity (Spearman rank correlation) than between the opposite sexes. Collectively, the observed sex-dimorphic metabolic transcript profiles suggest that sex is a main factor that plays an important role in regulating expression of metabolic genes in response to various chemical perturbations. Furthermore, the correlation strength of the metabolic transcripts within the same sex group increases with prolonged duration of chemical treatment.

### Identification of Sex-dimorphically Expressed Metabolic genes by a Devised Scoring Scheme

Next, we investigated if chemical perturbations might induce converged metabolic states in a sex-dependent manner through common metabolic genes and pathways, as it has been previously reported that there is a common mechanism in toxicant-induced inhibition of signaling pathway [27]. In order to identify metabolic genes that commonly display sex-dimorphic response under different chemical perturbations, we developed a sex-dependent expression score (SDES) with a numerical value between -1 to 1 for each gene to evaluate the magnitude of gene response acting in opposite directions in male and female fish (for details, see **Materials and Methods**). High positive SDES (0.5–1.0) indicates genes with strong sex-biased transcriptional activity during various chemical perturbations, i.e. these genes are oppositely responded to all chemical perturbations between male and female fish (**Figure S3**). On the contrary, negative SDES means the gene response is not sex-biased. The ranked lists of genes and corresponding SDES at four time points are presented in **Table S3**.

**Figure 4:**
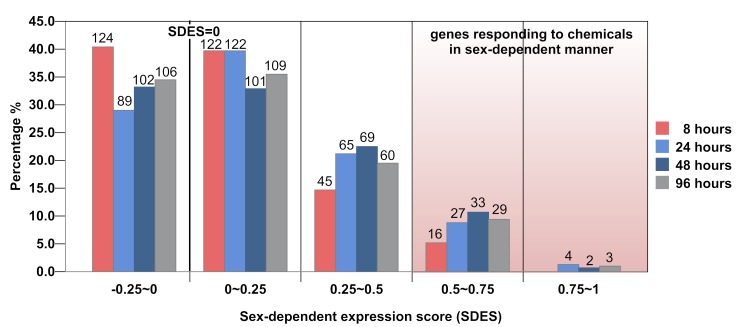
Distribution of metabolic genes according to their sex-dependent expression score (SDES). The histogram shows the distribution of metabolic genes according to their sex-dependent expression score (SDES) at the four time points of chemical perturbation. Each vertical bar represents the percentage of genes with SDES in a specific range. Genes with sex-dimorphic expression are defined as those associated with SDES equal or greater than 0.5 (SDES≥0.5). The number of genes for each group is indicated on top of each bar.

The distribution of metabolic genes according to their SDES was plotted in a histogram (**Figure 4**). It is apparent that a large number of metabolic genes were associated with positive SDES, suggesting their commonality in sex-dependent responses to chemicals. We defined genes with SDES higher than 0.5 as those commonly responding to chemicals in a sex-dependent manner. Overall, they corresponded to 5.2%, 9.8%, 11.1%, and 10.4% of total metabolic genes in our study at 8, 24, 48, and 96 hours, respectively. These percentages are in good accordance with the correlation strength of metabolic transcript profiles within the same sex presented in **Figure 3B**, suggesting that the number of sex-dimorphically expressed genes contribute significantly in shifting the correlation strength of metabolic transcript profiles within the same sex group.

**Figure 5:**
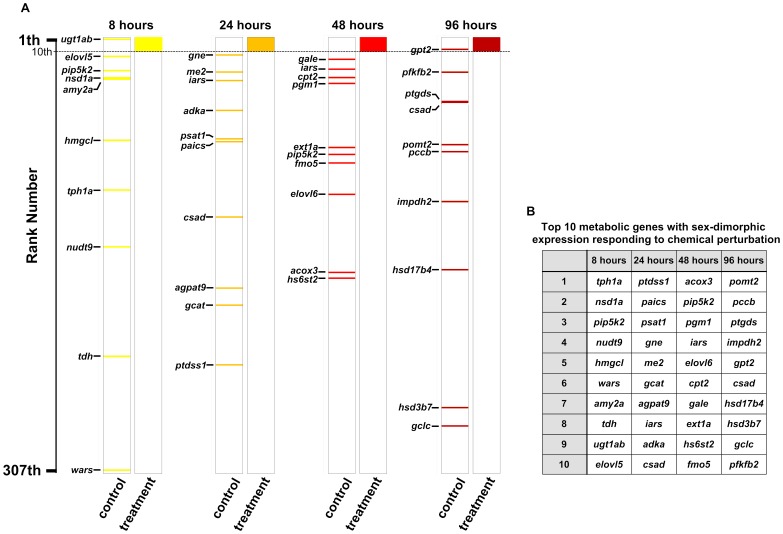
Comparison of sex-dimorphically expressed metabolic genes between chemical treated and control (untreated) groups. (A) Distribution of top ten sex-dimorphically expressed metabolic genes in response to chemical perturbation against ranked list of the control (untreated) groups. The top ten genes are generally randomly distributed throughout the ranking lists. (B) The identity of the top 10 sex-dimorphically expressed metabolic genes in the chemical treated groups at the four different time points.

To investigate whether the observed differences in gene activity between the opposite sexes existed under normal physiology, we also compared the gene expression levels between male and female in the control (untreated) groups. We performed Student’s *t*-test to examine the metabolic transcript profile between male and female fish at 8 h, 24 h, 48 h, and 96 h. The metabolic genes were then ranked according to their statistical significance (p-value) in decreasing order of sexual dimorphism as shown in **Table S4**. We compared the ranked lists from the chemical treated (**Table S3**) and untreated groups (**Table S4**) to determine whether there are distinguishable sex-dimorphic gene expression profiles induced by chemicals. We found that most of the top-ranked genes expressed in the chemical treatment group are located at the middle or the bottom of the ranked gene list in the untreated group (**Figure 5**). This further indicates that the sex-dimorphic expression of metabolic genes identified in the chemical treatment groups are indeed induced by chemical perturbations and are not merely part of the existing molecular differences between the two sexes.

**Figure 6:**
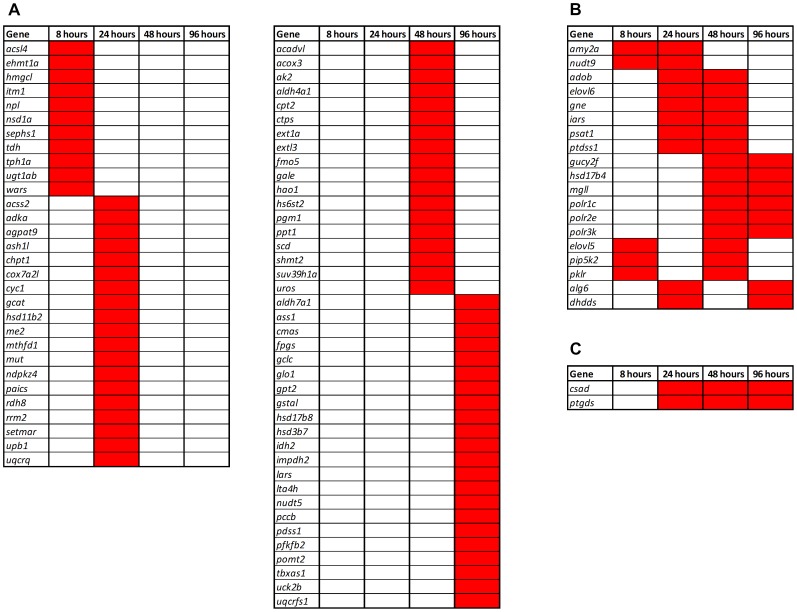
Sex-dimorphically expressed metabolic genes in response to chemical perturbation at different time points. All sex-dimorphically expressed metabolic genes were selected based on SDES of 0.5 or above. Genes that are sex-dimorphically expressed at one, two or three time points are presented in (A), (B) and (C) respectively. No gene is consistently behaved sex-dimorphically at all four time points.

Genes commonly responding to different chemicals in a sex-dependent manner (SDES ≥0.5) and their corresponding time points are shown in **Figure 6**. We noted that transcriptional activities of many xenobiotic metabolism related genes became sex-biased upon chemical perturbation, for example, *fmo5 (*flavin containing monooxygenase), *aldh4a1* and *aldh7a1* (aldehyde dehydrogenase), *gstal* (glutathione S-transferase), and *ugt1ab* (UDP glucuronosyltransferase). The cytochrome P450 enzyme gene (*cyp1a*) also had a relatively high SDES score (SDES = 0.414) at 24 hours, indicating its sex-dimorphic responses to chemical treatments. Other than genes encoding xenobiotic metabolizing enzymes, several functionally diverse metabolic genes also showed sex-dimorphic responses, such as those encoding for ELOVL fatty acid elongase (*elovl*), DNA-directed RNA polymerase (*polr*), and hydroxysteroid dehydrogenase (*hsd*).

Furthermore, among the 91 metabolic genes that showed sex-dimorphic responses during chemical perturbations, 21 genes (23.1%) were present at two or more time points and 70 genes (76.9%) were present only at one specific time point (**Figure 6**). The temporal difference in sex-dimorphic expression suggests that both time-invariant and time-variant sex-dependent regulations are required to fine-tune metabolic homeostasis upon chemical perturbations.

### Identification of Enriched Sex-dimorphic Metabolic Pathways

As the functional units of metabolic systems are composed of metabolic pathways, it is also interesting to identify chemical-responsive sex-dimorphic pathways. Thus, we compared, by Gene Set Enrichment Analysis (GSEA) [28], the ranked lists of metabolic genes (**Table S3** for chemical treatment and **Table S4** for control group) with defined knowledge-based gene sets in metabolic pathways available in KEGG (for details, see **Materials and Methods**). Metabolic pathways, that were statistically significant (nominal p-value ≤0.05) and moderately (0.05≤ nominal p-value ≤0.1) enriched with sex-dimorphically expressed genes for chemical-treated and untreated groups at four time points are shown in **Table 1** and **Table 2**, respectively. The magnitude of sex-dependent activity for each pathway was indicated by normalized enrichment score (NES). A metabolic pathway with positive NES implies the presence of sex-dimorphic response during chemical perturbation. Negative NES implies no sex-dimorphic response associated with the pathway. The detailed lists of the NES and nominal p-values to each metabolic pathway are presented in **Table S5** and **Table S6**, respectively.

**Table 1:** Sex-dimorphic metabolic pathways under chemical treatments.

Time Point	Pathway	NES	Nominal p-value
8 hours	**Biosynthesis of unsaturated fatty acids**	1.64	0.005
	**Lysine degradation**	1.44	0.034
	Starch and sucrose metabolism	1.39	0.066
	Tryptophan metabolism	1.33	0.099
24 hours	**Glycerophospholipid metabolism**	1.67	0.003
	**Steroid hormone biosynthesis**	1.42	0.034
	Glycine, serine and threonine metabolism	1.37	0.071
	Drug metabolism – other enzymes	1.37	0.075
48 hours	**Purine metabolism**	1.41	0.015
	**Fatty acid metabolism**	1.41	0.031
	**Amino sugar and nucleotide sugar metabolism**	1.40	0.038
	**Pyrimidine metabolism**	1.37	0.044
	Biosynthesis of unsaturated fatty acids	1.38	0.062
96 hours	**Drug metabolism – other enzymes**	1.43	0.038
	Glycerolipid metabolism	1.36	0.070
	Pyrimidine metabolism	1.31	0.075

Listed are significantly (nominal p-value ≤0.05; highlighted in bold) and moderately (0.05≤ nominal p-value ≤0.1) enriched metabolic pathways. NES: normalized enrichment score.

**Table 2:** Sex-dimorphic metabolic pathways in control (untreated) group.

Time Point	Pathway	NES	Nominal p-value
8 hours	N-glycan biosynthesis	1.40	0.052
	Lysine degradation	1.34	0.059
	Ether lipid metabolism	1.37	0.064
24 hours	**Propanoate metabolism**	1.31	0.040
48 hours	**Selenoamino acid metabolism**	1.51	0.009
	**Propanoate metabolism**	1.39	0.018
	Valine, Leucine and Isoleucine biosynthesis	1.33	0.070
	Steroid hormone biosynthesis	1.32	0.074
	Glycerolipid metabolism	1.27	0.088
96 hours	**Citrate cycle (TCA Cycle)**	1.52	0.005
	**Alanine, aspartate and glutamate metabolism**	1.45	0.017
	**Propanoate metabolism**	1.38	0.025
	**Ether lipid metabolism**	1.41	0.031
	**Selenoamino acid metabolism**	1.37	0.044
	**Pentose and glucuronate interconversions**	1.36	0.048
	Arachidonic acid metabolism	1.34	0.067
	Tyrosine metabolism	1.31	0.074

Listed are significantly (nominal p-value ≤0.05; highlighted in bold) and moderately (0.05≤ nominal p-value ≤0.1) enriched metabolic pathways. NES: normalized enrichment score.

As shown in **Table 1** and **Table 2**, enriched sex-dimorphic pathways are different between chemical-treated and untreated groups, suggesting that chemicals indeed induced different sets of sex-dimorphic pathways. As there was no feeding during the 96-hour chemical exposure experiments for both treated and untreated groups, sex-dimorphic pathways identified in the control (untreated) groups mainly corresponded to responses to non-feeding condition between males and females. Alterations of sex-dimorphic gene expression and pathways during non-feeding or manipulation of dietary carbohydrate have also been observed in a previous study [18].By comparing two pathway lists in **Table 1** and **Table 2**, we noticed that several pathways, including drug metabolism-other enzymes, biosynthesis of unsaturated fatty acids, fatty acid metabolism, glycerophospholipid metabolism, and purine and pyrimidine metabolism, showed sex-dimorphic responses only under chemical treatments. The enrichment of drug metabolism-related pathway at 24 and 96 hours indicates that a large portion of genes involving in xenobiotic metabolism process responded differentially in male and female fish. This also suggests that zebrafish has sex-dependent capabilities of xenobiotic detoxification under chemical treatments. As the sexual differences of xenobiotic metabolism has also been reported in mammals [15], [16], [21], these observations might imply common sex-dependent xenobiotic responses between fish and mammals. In addition, sex hormones and other non-hormonal sex-factors are known to regulate lipid-related and nucleotide-related metabolism [29]–[31]. The relevant pathways are also sex-dimorphic under toxicopathological homeostasis in our study. In view of the significance of biological functions mediated by these pathways during xenobiotic encountering [32], [33] it is important to consider the sex factor as a variable in relevant studies.

### Network Analysis Revealed Preferential Enrichment at Lipid and Nucleotide Metabolisms with Prolonged Chemical Perturbations

**Figure 7:**
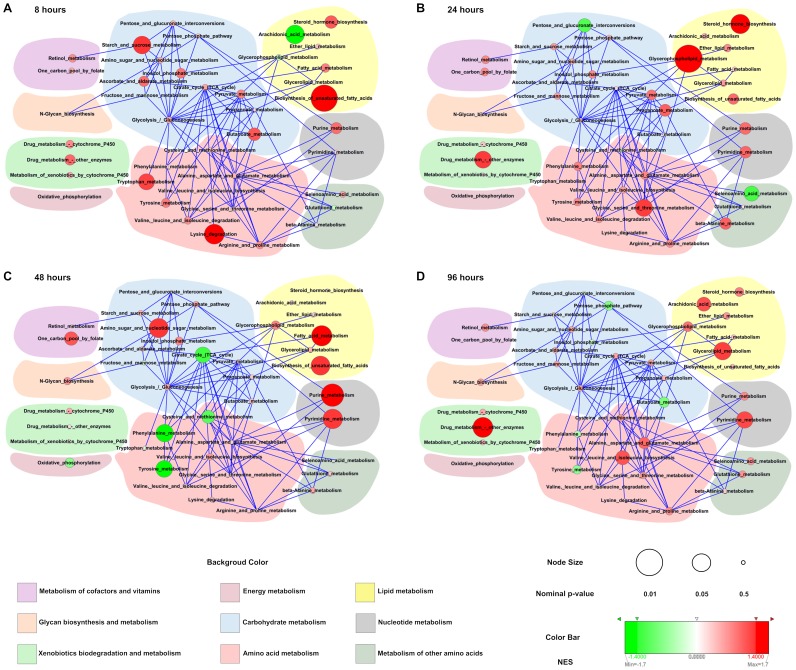
Network of metabolic pathways with sex-dimorphic responses to chemical perturbation in zebrafish liver. A node represents metabolic pathway and edges denote cross-talks between pathways. Size of node denotes the statistical significance (nominal p-value) of the metabolic pathway and color represents the normalized enrichment score (NES) score from Gene Set Enrichment Analysis (GSEA) analysis. The background color represents higher level of functional categories as indicated by the color code at the bottom of the figure. Panels A-D displays networks at 8, 24, 48 and 96 hours respectively.

In order to understand how sex-dimorphic pathways are coordinated at the systems level, the zebrafish metabolic pathway network was constructed using KEGG-derived metabolic pathways, with minimum 5 genes detected in microarray experiments for a given pathway (**Figure 7**). The resulting network consists of 41 nodes (metabolic pathways) and 84 edges. Two pathways are connected if a product of one pathway is utilized as a substrate in another pathway (for details, see **Materials and Methods**). The size of each node is proportional to log_10_(1/nominal p-value), where nominal p-value is the indicator of statistical significance of pathway in GSEA analysis, and the color denotes the normalized enrichment score (NES) (see **Table S5** and **Table S6** for the nominal p-value and NES scores of all pathways in chemical treatment and control condition respectively). A large red node represents statistically enriched (p-value <0.05 in GSEA analysis) sex-dimorphic responsive pathways, where a small node indicates the lack of statistical significance, and a green node means there is no sex-dimorphic response associated with the pathway.

At early stage of chemical perturbations (8 and 24 hours), statistically enriched sex-dimorphic metabolic pathways were distributed over various metabolic categories, such as carbohydrate metabolism, amino acid metabolism, lipid metabolism, and xenobiotics biodegradation and metabolism, as indicated by the scattered distribution of large red nodes all over the networks, reflecting sex-dependent response of numerous metabolic processes (**Figure 7A and 7B**). At late stage of chemical perturbation (48 and 96 hours), most significantly enriched pathways belonged to lipid metabolism and nucleotide metabolism (**Figure 7C and 7D**), which suggests that progressive chemical perturbation induced sex-dependent response on many specific metabolic functions rather than on broad metabolic processes as oppose to earlier time points.

Taken together, our network analysis indicates sex-dimorphic metabolic pathways are differentially distributed across different functional modules during chemical treatments. This suggests the existence of different regulatory modes controlling the activities of metabolic pathways in opposite sexes during homeostatic responses to chemical perturbation. These sex-dependent regulations of metabolic functions can subsequently lead to differential cellular signaling events to regulate downstream biological processes in response to extended chemical insults.

### Conclusion

Our toxicogenomic analysis revealed common sex-dependent metabolic gene expression in response to chemical insults in the zebrafish liver. The chemical-induced sexual dimorphism in metabolic processes can be observed not only at the gene and pathway level, but also at the higher network level with defined functional modules. Moreover, the affected sex-dimorphic metabolic pathways changed dynamically during the chemical perturbation period, with most genes exhibit temporal sex-dependent behavior, suggesting homeostatic and feedback adjustments. The present findings of sex-dimorphic responses to chemical insults in male and female fish warrant further investigations in mammals as they may have toxicological, liver pathological and metabolic disorder implications. Therefore, our study underscores the importance of sex factor in both toxicological and biomedical researches.

## Materials and Methods

### Zebrafish

Adult zebrafish (6 months –1 year old) were obtained from a local fish farm. The fish were allowed to acclimatize in aquaria for several days before transferred into smaller tanks for chemical exposure. Male and female fish were distinguished based on typical morphological differences including the size/shape, body color, abdomen etc. [34]. The gonads were also readily distinguishable by the naked eye during sampling of the liver tissues. All experimental protocols were approved by Institutional Animal Care and Use Committee (IACUC) of National University of Singapore (Protocol 079/07).

### Chemical Treatments

Adult male and female fish were treated in separate tanks. In each chemical treatment, 60 male and 60 female fish were maintained at a density of one fish per 200 ml for sampling at four time-points (8-hour, 24-hour, 48-hour, and 96-hour). As the liver is a major organ in metabolism, the fish were not fed throughout the experiments in order to minimize interfering effects due to food metabolism that could confound metabolic responses elicited by chemicals. For each time-point group, 3 pooled liver samples were obtained with each pool containing livers from 4 individual fish, hence 12 fish were used for biological samples in each time-point group and a total of 48 fish were used for each chemical treatment for one sex. Concentrations of chemicals and number of replicates used are presented in **Table S1**. The concentrations of the chemical used in the study caused 10–20% mortality based on 96-hour acute toxicity tests carried out under similar conditions. The concentrations were chosen as they were sufficient to induce toxicity with minimal mortality (10–20%), hence leaving enough samples for the microarray experiments. Further details of chemical treatments had been described in our previous works [35], [36].

### Microarray Experiments and Data Processing

Total RNA was extracted using Trizol reagent (Invitrogen, USA) according to the manufacturer’s instructions. Reference RNA for microarray hybridization was obtained by pooling total RNA from whole adult male and female zebrafish at 9∶1 ratio. The 9∶1 male:female ratio was used to reduce some of the highly abundant female RNA transcripts (e,g, *vitellogenin)* which otherwise could easily saturated the hybridization signals on these feature probes by the reference RNA alone. Here we used a common reference design in a two-color dye microarray system and we found that a 9∶1 male:female ratio were able to provide sufficient reference hybridization signal with minimal saturated feature probes on the arrays [37]. The integrity of RNA samples was verified by gel electrophoresis, and their concentrations were determined by UV spectrophotometer. Reference RNA was co-hybridized with RNA samples either from chemically treated or control fish on a glass array spotted with 16.5K zebrafish oligo probes. Both reference and sample RNAs were reverse-transcribed and labelled differently using fluorescent dyes Cy-3 or Cy-5. After hybridization at 42°C for 16 hours in hybridization chambers, the microarray slides were washed in a series of washing solutions (2X SSC with 0.1% SDS; 1X SSC with 0.1% SDS; 0.2X SSC and 0.05X SSC; 30 seconds each), dried by low-speed centrifugation and scanned for fluorescence detection using the GenePix 4000B microarray scanner (Axon Instruments).

The raw microarray data was normalized using Lowess method [38] in the R package (http://www.braju.com/R/). The zebrafish genes were mapped to human homologous genes as described previously [37] and was subsequently used to map with metabolic genes defined in the Kyoto Encyclopedia of Genes and Genomes (KEGG) database [26]. Genes missing from microarray data under one or more chemical-treatment conditions were eliminated. Student’s *t*-test was performed to evaluate statistical significance between treatments and controls. Each gene was assigned with the value of log_10_(1/P) where P is the p-value of a gene obtained from Student’s t-tests. Z-score normalization [39] was performed to standardize the value of log_10_(1/P) for each gene with respect to each chemical treatment to enable the comparison of microarray data generating from different experiments (**Table S2**). All microarray data generated is MIAME compliant. The raw data used in this study have been deposited in Gene Expression Omnibus (GEO) and the accession numbers are given in **Table S1**.

To assess gene coverage of our microarray platform (GPL2715), we compared the number of annotated genes in our platform with those in recently updated platforms and we found 80.9% of well annotated genes in our platform were included in GPL15450 (updated on Apr 26, 2012), 79.6% in GPL9907 (updated on Dec 20, 2011), and 80% in GPL14688 (updated on Oct 08, 2011). The comparison results suggest that genes in our microarray platform are well covered.

### Hierarchical Clustering

The software ‘Cluster’ [40] (http://www.eisenlab.org/eisen/?page_id=42) was used to perform hierarchical clustering analysis. Spearman Rank Correlation and Pearson Correlation were applied to calculate similarity metric; average linkage was applied as clustering method. The tree image of profiles clustering and the heat maps in **Figure 2**, **Figure 3A**, **Figure S1**, and **Figure S2** were generated by ‘TreeView’ (http://www.eisenlab.org/eisen/?page_id=42).

### Sex-dependent Expression Score (SDES)

The purpose of the SDES was to evaluate the magnitude of gene response acting in opposite directions in male and female fish under different chemical perturbation. The principle was based on assessing the compactness of individual gene transcriptional activities under different chemical treatments within the same sex group versus the isolations between opposite sex groups. The basic concept of SDES is illustrated in **Figure S3**.

Grouping expression data 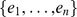 of 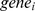 under 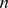 conditions into male group and female group 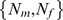, where 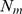 and 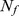 denotes the number of conditions involving male and female respectively, the sex-dependent expression score (SDES) for 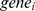 is
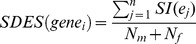
where 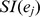 is the *Silhouette index* of 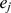. Assuming 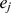 belongs to male group, then the *Silhouette index* of 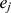 contains two elements: (i) compactness 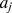 which describes the average distance of 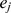 to all other data points in the same cluster



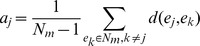
(ii) isolation 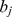 which describes the average distance between 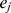 to the members of female group,



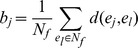
then the *Silhouette index* of 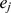 is defined by



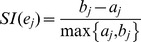

*Silhouette index* of 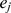, 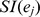, ranges from +1 to −1. Negative values indicate that this data should belong to a different cluster rather than the computed one. Therefore, the score of 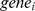 ranges from +1 to −1, and a higher score indicates significant sex-dependent activities of 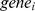 under diverse chemical treatments.

### Gene Set Enrichment Analysis (GSEA)

Gene Set Enrichment Analysis (GSEA) [28] is a computational method that determines whether a pre-defined gene set is over-represented at the top or bottom ranking of whole transcriptome profile. In our study, the *a priori* defined set of metabolic genes were those involving in the same metabolic pathway defined by KEGG database [26]. The ranked lists of metabolic genes were given in **Table S3** (chemical treatments) and **Table S4** (control and untreated). An enrichment score (ES) for each pathway was calculated by walking down the ranked gene list, increasing a running-sum statistic when a gene in a pre-defined gene set was encountered and decreasing it when the gene was absent. The ES is the maximum deviation from zero encountered in the random walk corresponding to a weighted Kolmogorov-Smirnov-like statistic [41]. The statistical significance of a given ES was estimated by using permutation test procedure (10,000 permutations used). The ES of the gene set for the permuted data were recomputed to generate a null distribution for the ES. The empirical, nominal p-value of the observed ES was then calculated in relative to this null distribution. The ES for each gene set was first normalized to the size of the set yielding a normalized enrichment score (NES) with the following relation:

NES = actual ES/mean ES (calculated from all permutations of the dataset).

In our study, metabolic pathways which have minimum 5 genes in the gene list (**Table S5** and **Table S6**) were considered for GSEA calculation.

### Metabolic Pathway Network Construction

In the metabolic pathway network, each pathway is represented as node. If the product of one pathway is used as the substrate of another pathway, there will be a link connecting these two pathways. For each pathway, its related partners can be found from KEGG database [26] (ftp://ftp.genome.jp/pub/kegg/pathway/pathway). In our study, only those metabolic pathways involving in GSEA calculation were used for network construction. The constructed metabolic pathway network has 41 nodes and 84 links.

## Supporting Information

Figure S1 Hierarchical clustering of metabolic transcript profiles (Spearman rank correlation) at the four time points of chemical perturbations.
(TIF)

Figure S2 Hierarchical clustering of metabolic transcript profiles (Pearson correlation) at the four time points of chemical perturbations.
(TIF)

Figure S3 Basic principle of sex-dependent expression score (SDES). The design principle of sex-dependent expression score (SDES) is to evaluate the magnitude of gene response acting in opposite directions in male and female fish under different chemical perturbations. A red box represents up-regulation and a green box represents down-regulation. (A) Gene 1 has similar expression trend within male group (up-regulation) and female group (down-regulation) respectively, but, dissimilar between two sexes. In this situation, a high SDES value is assigned to gene 1. (B) Gene 2 has no clear pattern of sex-dependent gene expression trend of its expression is inconsistent within one sex and thus gene 2 has a low SDES.
(TIF)

Table S1 Categories and types of hepatic transcriptome data used in this study. The table summarizes the liver samples used in the present study and their GEO series accession numbers for microarray data.
(DOC)

Table S2 Normalized Z-score of metabolic genes for their expression levels. The table summarizes the level of metabolic gene expression in all samples used in the study.
(XLS)

Table S3 Ranked lists of metabolic genes according to sex-dependent expression score (SDES) under chemical treatment. The table includes ranked lists of metabolic genes according to SDES for all four time points of the chemical treatment groups.
(XLS)

Table S4 Ranked lists of metabolic genes according to the p-value (Student’s t-test) of sexual dimorphism in control (untreated) group. The table includes ranked lists of metabolic genes according to the p-value (Student’s t-test) in the control (untreated) groups at the four time points.
(XLS)

Table S5 Gene Set Enrichment Analysis (GSEA) results of enriched sex-dimorphic metabolic pathways under chemical treatment. The table contains four panels of Gene Set Enrichment Analysis (GSEA) results of enriched sex-dimorphic metabolic pathways under chemical treatment.
(XLS)

Table S6 Gene Set Enrichment Analysis (GSEA) results of enriched sex-dimorphic metabolic pathways in control (untreated) group. The table contains four panels of Gene Set Enrichment Analysis (GSEA) results of enriched sexually dimorphic metabolic pathways in control (untreated) group.
(XLS)
