# Supplementary figures and images for: Toxicogenomic Analysis Suggests Chemical-Induced Sexual Dimorphism in the Expression of Metabolic Genes in Zebrafish Liver
Source: PLoS One. 2012 Dec 18;7(12):e51971. doi: 10.1371/journal.pone.0051971 (PMC3525581; doi:10.1371/journal.pone.0051971)

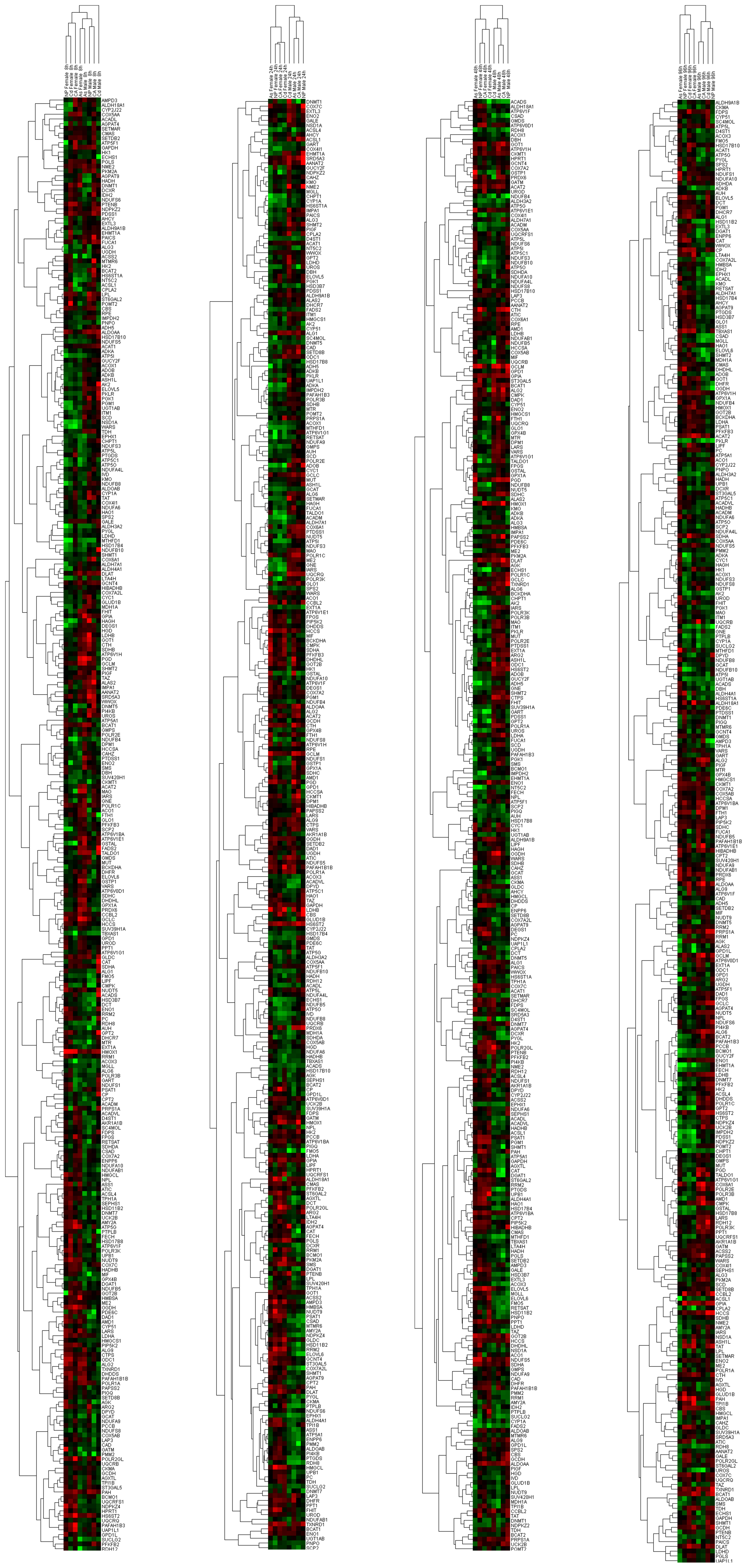

Supplement: Figure S1 — Hierarchical clustering of metabolic transcript profiles (Spearman rank correlation) at the four time points of chemical perturbations. (TIF) [file pone.0051971.s001.tif]

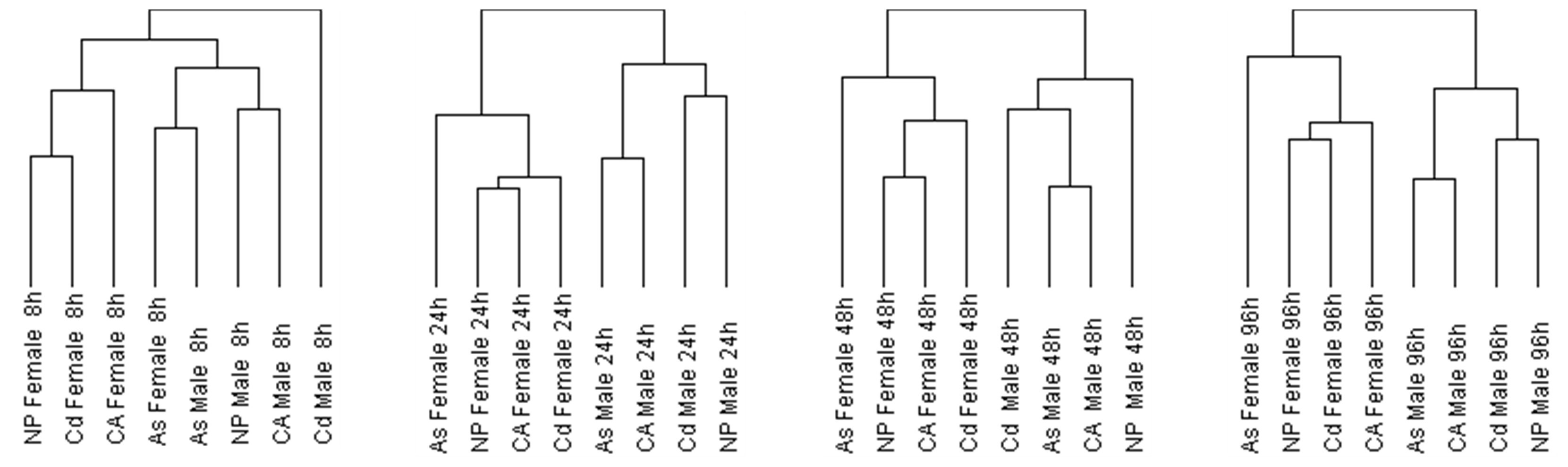

Supplement: Figure S2 — Hierarchical clustering of metabolic transcript profiles (Pearson correlation) at the four time points of chemical perturbations. (TIF) [file pone.0051971.s002.tif]

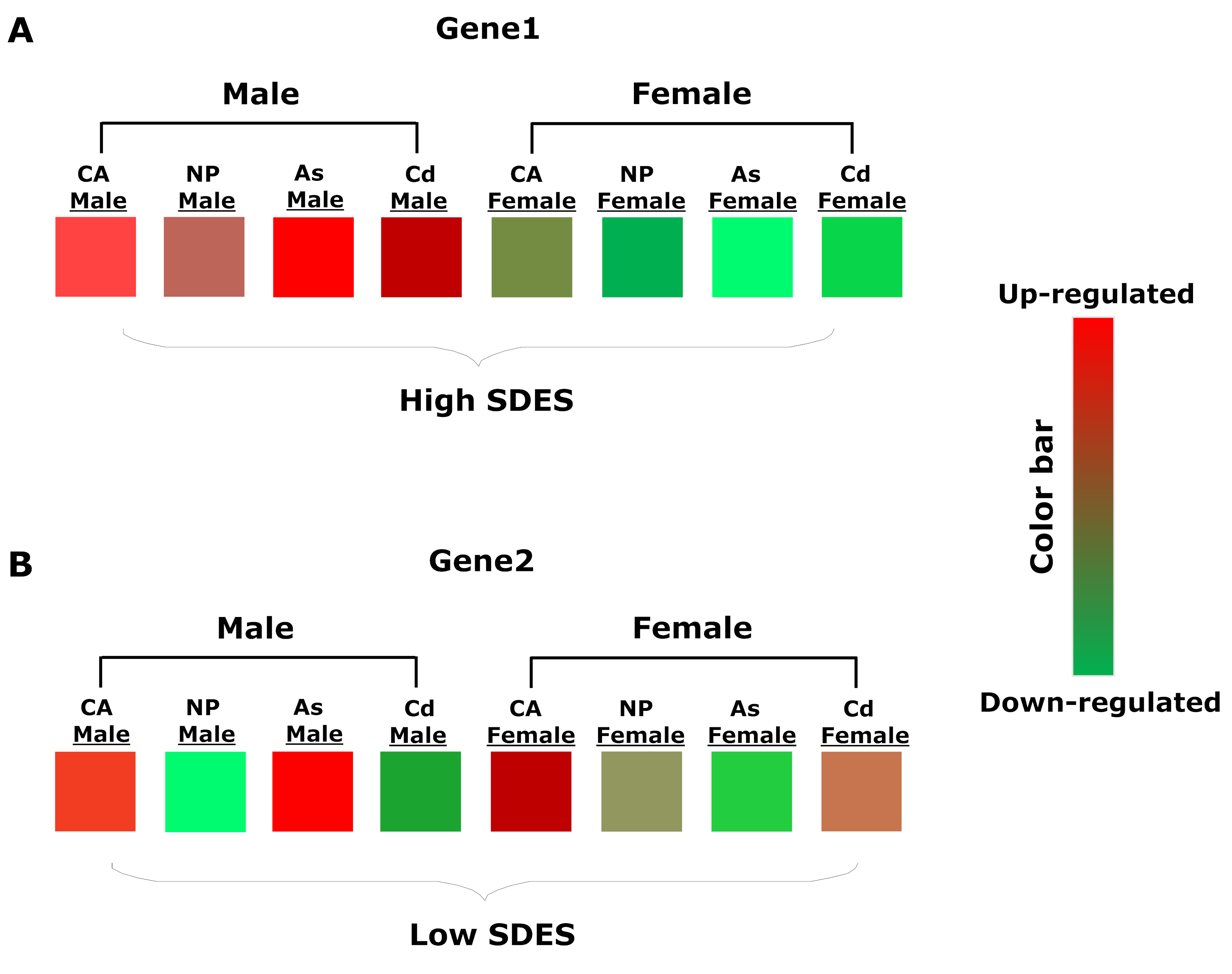

Supplement: Figure S3 — Basic principle of sex-dependent expression score (SDES). The design principle of sex-dependent expression score (SDES) is to evaluate the magnitude of gene response acting in opposite directions in male and female fish under different chemical perturbations. A red box represents up-regulation and a green box represents down-regulation. (A) Gene 1 has similar expression trend within male group (up-regulation) and female group (down-regulation) respectively, but, dissimilar between two sexes. In this situation, a high SDES value is assigned to gene 1. (B) Gene 2 has no clear pattern of sex-dependent gene expression trend of its expression is inconsistent within one sex and thus gene 2 has a low SDES. (TIF) [file pone.0051971.s003.tif]
