# Supplementary material for: Toxicogenomic Analysis Suggests Chemical-Induced Sexual Dimorphism in the Expression of Metabolic Genes in Zebrafish Liver
Source: PLoS One. 2012 Dec 18;7(12):e51971. doi: 10.1371/journal.pone.0051971 (PMC3525581; doi:10.1371/journal.pone.0051971)
Supplement: Table S1 — Categories and types of hepatic transcriptome data used in this study. The table summarizes the liver samples used in the present study and their GEO series accession numbers for microarray data. (DOC) [file pone.0051971.s004.doc]

**Table S1. Categories and types of hepatic transcriptome data used in this study.** CA = 4-Chloroaniline; NP = 4-Nitrophenol; As = Arsenic (V); Cd = Cadmium (II)

Sample Label Treatment Conditions GEO Series Accession #of replicates

CA_8h_Female 20 mg/L, 8h, female fish GSE30057 3

CA_24h_Female 20 mg/L, 24h, female fish GSE30057 3

CA_48h_Female 20 mg/L, 48h, female fish GSE30057 3

CA_96h_Female 20 mg/L, 96h, female fish GSE30057 3

CA_8h_Male 20 mg/L, 8h, male fish GSE30055 3

CA_24h_Male 20 mg/L, 24h, male fish GSE30055 3

CA_48h_Male 20 mg/L, 48h, male fish GSE30055 3

CA_96h_Male 20 mg/L, 96h, male fish GSE30055 3

NP_8h_Female 7 mg/L, 8h, female fish GSE30060 3

NP_24h_Female 7 mg/L, 24h, female fish GSE30060 3

NP_48h_Female 7 mg/L, 48h, female fish GSE30060 3

NP_96h_Female 7 mg/L, 96h, female fish GSE30060 3

NP_8h_Male 7 mg/L, 8h, male fish GSE30058 3

NP_24h_Male 7 mg/L, 24h, male fish GSE30058 3

NP_48h_Male 7 mg/L, 48h, male fish GSE30058 3

NP_96h_Male 7 mg/L, 96h, male fish GSE30058 3

As_8h_Female 15 ppm (~192 µM), 8h, female fish GSE30062 3

As_24h_Female 15 ppm (~192 µM), 24h, female fish GSE30062 3

As_48h_Female 15 ppm (~192 µM), 48h, female fish GSE30062 3

As_96h_Female 15 ppm (~192 µM), 96h, female fish GSE30062 3

As_8h_Male 15 ppm (~192 µM), 8h, male fish GSE3048 3

As_24h_Male 15 ppm (~192 µM), 24h, male fish GSE3048 3

As_48h_Male 15 ppm (~192 µM), 48h, male fish GSE3048 3

As_96h_Male 15 ppm (~192 µM), 96h, male fish GSE3048 3

Cd_8h_Female 30 μg, 8h, female fish GSE41622 2

Cd_24h_Female 30 μg, 24h, female fish GSE41622 3

Cd_48h_Female 30 μg, 48h, female fish GSE41622 3

Cd_96h_Female 30 μg, 96h, female fish GSE41622 2

Cd_8h_Male 30 μg, 8h, male fish GSE41623 2

Cd_24h_Male 30 μg, 24h, male fish GSE41623 2

Cd_48h_Male 30 μg, 48h, male fish GSE41623 3

Cd_96h_Male 30 μg, 96h, male fish GSE41623 3
